# NIST Polymer Pyrolysis Search: A New Pyrolysis GC–MS Search Program and Mass Spectral Reference Library

**DOI:** 10.1021/acs.est.5c18872

**Published:** 2026-06-29

**Authors:** Edward P. Erisman, Yamil Simón-Manso, William E. Wallace

**Affiliations:** Mass Spectrometry Data Center, Biomolecular Measurement Division, 10833 National Institute of Standards and Technology, Gaithersburg, Maryland 20899, United States

**Keywords:** py−GC/MS, mass spectral reference library, microplastics, nanoplastics, pyrolysis

## Abstract

Pyrolysis is the thermal decomposition of a material in an inert environment and is used in combination with gas chromatography–mass spectrometry (py–GC/MS) for analyzing the chemical composition of a wide variety of organic materials. The identification and deconvolution of polymers, including mixtures with complex matrixes using a searchable mass spectral library of individual pyrolysis products (pyrolyzates) is presented. It is demonstrated that this method can successfully identify components in polymer mixtures, including polyolefins, a mixture of polymers that have secondary reactions during pyrolysis, a copolymer as distinct from homopolymers of its individual components, and a polymer within a complex biological matrix (with a prepyrolysis in situ hydrolysis and methylation step). The resulting interactive, freely available search software application and pyrolysis reference library for the qualitative identification of polymers is described.

## Graphical abstract

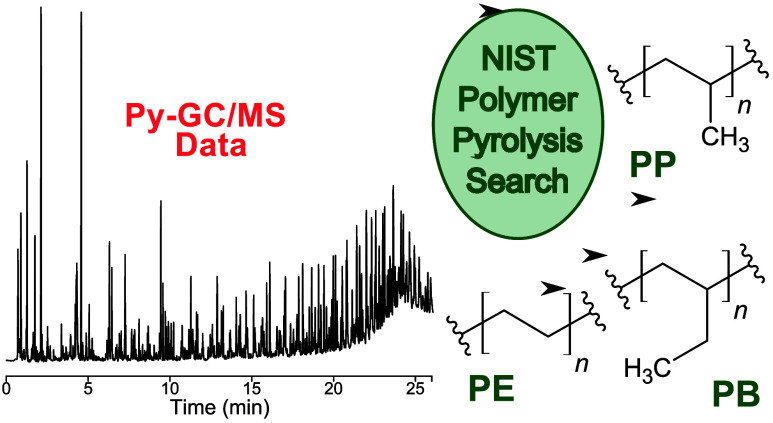

## 1 Introduction

Chemical analysis of polymers, including copolymers and polymer blends, is challenging particularly when they are present in low concentrations and as small particles in complex matrices. This is due to a variety of factors, including high molecular weights, low volatilities, different solubilities, and varying responses to temperature change. However, accurate and confident identification of polymers is essential for industry, healthcare, and environmental sciences. Polymer analysis can be destructive or nondestructive. Nondestructive methods include optical spectroscopy techniques.^1–5^ Most prominent among destructive methods are mass spectrometry^6–9^ often coupled with a decomposition step. In particular pyrolysis–MS,^10^ and pyrolysis–GC/MS^11^ (py–GC/MS) play an important role in polymer analysis. Decomposition can be achieved thermally in an inert environment (pyrolysis) or chemically (such as by hydrolysis,^12^ thermally assisted hydrolysis/methylation (THM),^13–15^ or alkali fusion^16^) either prior or in-line with the instrument. Degradation such as hydrolysis, THM, or alkali fusion could be part of a scheme to remediate complex matrix effects or for polymer identification. A comprehensive review detailing the fundamentals of py–GC/MS, specifically focusing on its application in environmental analysis, organic matter characterization and microplastic identification can be found in the literature.^17^ Also, fundamental applications and industrial uses are well documented.^18,19^ The technique’s ability to qualitatively and quantitatively identify plastics within solid multicomponent blends (e.g., combinations of polyethylene, polypropylene, polystyrene, polyamide, and polycarbonate) by isolating their characteristic pyrolysis products has also been shown.^20^ While py–GC/MS is a long-standing technique used to identify polymers, recently it has been pointed out that significant challenges remain for the accurate analysis of difficult samples, such as microplastics in complex biological matrices.^21,22^

Confidence and ease of accurate polymer identification is greatly aided by using reference libraries. Pyrolysis libraries generally fall into three categories: chromatogram-based,^23^ combined^18,24^ or direct^25^ mass spectral-based, and marker compound-based.^26^ Chromatogram-based searches do not use mass spectral information. Instead, they make a match based on the shape of the chromatogram. This is much more difficult when a sample contains multiple polymers and/or is in a complex matrix. Direct or combined mass spectral-based searches do not use chromatographic information. Either there is no chromatographic separation (such as py–MS), or the mass spectra of the chromatographic peaks are extracted and summed together. Using this method, it is difficult to deconvolute polymer mixtures and/or complex matrices. Commercial pyrolysis search software exists using this method. Marker compound-based^24^ libraries rely on identifying one, or ideally more, unique pyrolyzate(s) to identify the polymer. Knowledge of which pyrolyzate(s) to count as a marker compound is essential to the performance of this technique because a specific chromatographic peak could arise from the pyrolysis of different polymers, or the pyrolysis of a matrix component, or be part of the matrix itself. ASTM International standard D 8401 ^27^ could be considered as an example of this method although the identification of individual marker compounds is accomplished by specific fragment ions and therefore is not as robust as whole spectrum matching.

The NIST Mass Spectrometry Data Center generates mass spectral libraries and search software to interact with this standard data to aid users in identifying compounds.^28^ This py–GC/MS work is part of a broader focus on generating mass spectral libraries for plastics and plastic related compounds.^29^ Herein we describe a different approach to py–GC/MS data analysis by implementing a pyrolyzate compound library and retention index (RI) constrained library search to putatively identify the marker compounds in an automated fashion for the attribution of the parent polymer. The retention index of an analyte relates its retention time to those of two standards (often *n*-alkanes) eluting before and after it. The retention index is roughly independent of experimental differences (column flow, oven temperature ramp rate, column phase ratio, etc.) for a given column phase allowing for more accurate chromatographic comparisons between systems. The aim is to create a scheme that easily identifies the polymers present in a mixture, is robust to changes in sample conditions, pyrolysis, and chromatography, and requires minimal user input. The result is a data analysis scheme and corresponding computer application, NIST Pyrolysis Polymer Search (NIST PPS) (https://chemdata.nist.gov/dokuwiki/doku.php?id=chemdata:polymersearch), that implements an RI constrained mass spectral library search that putatively identifies multiple marker compounds and attributes them to the parent polymer. Some general limitations of pyrolysis are only briefly mentioned and possible strategies to overcome those limitations are demonstrated to not preclude using this method of analysis. Since the result of this work is a software tool to aid in the analysis of pyrolysis data, no specific claim is made regarding what constitutes necessary and sufficient support for any conclusion derived from this work.

This work is an initial step toward confident qualitative identification. Correct qualitative identification is necessary before any attempts at quantitative analysis can be made. Both qualitative and quantitative analysis depend on the reproducibility of py–GC/MS which is beyond the scope of this work.

## 2 Materials and Methods

### 2.1 Material Selection, Sample Preparation, and py–GC/MS Method

The data used to create the associated pyrolysis mass spectral library was generated according to the following experimental variables. Polymer reference samples were either from Scientific Polymer Products Inc. polymer sample kit (catalog # 205) or, in the case of polyethylene terephthalate (PET), individually sourced from Goodfellow Advanced Materials (ES30-PD-000132). A C7–C40 *n*-alkane standard (Sigma-Aldrich product number 49452-U) was used for retention index calculations. An off-the-shelf chocolate almond flavored whey protein powder drink mix (Rise Wellness—3.5 g total fat and 25 g protein per 35 g net powder per nutrition label) was dry mixed with polyethylene, and 25 wt % tetramethylammonium hydroxide (TMAH) in MeOH (Sigma-Aldrich product number 334901) was used to test a prepyrolysis hydrolysis/methylation step. Different alternatives for sample preparation were tested, but no single solution was found that worked for all materials. Polymers were manually cut to size (≈50 μg to 200 μg) with a knife or milled (room temperature or cooled with liquid nitrogen) using an IKA Tube Mill 100. Milling rpm, time, and frequency were adjusted for best outcome specific to the polymer. Samples were weighed using a microbalance (Radwag UYA 2.5Y). Some binary or ternary polymers mixtures were prepared, as well as a blend of polyethylene with protein powder to mimic a complex biological matrix. See Supporting Information S1 for more sample information.

A JEOL GC-Alpha GC–MS system (JEOL AccuTOF MS with an Agilent 8890 GC) coupled to a Gerstel pyrolysis system (PYRO—https://www.gerstelus.com/products-pyrolysis/) and Gerstel programmable temperature vaporizer inlet with liquid nitrogen cryo-trapping was used in this work. The Gerstel system has a pyrolysis filament within a thermal desorption unit (TDU) connected to the GC inlet liner by a short transfer line (liner-in-liner). The majority of this work used a ramped pyrolysis method, and the unit was operated in evolved gas mode (simultaneous temperature ramp of TDU and pyrolyzer at the same rate). The TDU was ramped from (90 to 350) °C at 5 °C/s (after a 0.5 min delay), the pyrolyzer was ramped from (100 to 800) °C at 5 °C/s (with no delay), the TDU transfer line was kept constant at 315 °C and the GC method was started 0.5 min after the pyrolyzer was turned off. Flash pyrolysis methods were used for comparison. The TDU was heated at 5 °C/s from (60 to 320) °C and held with the pyrolyzer turned on at the specified temperature (450, 500, 650) °C for 0.2 min and then turned off for 0.5 min after which the GC method was initiated. For both methods, the pyrolyzates were cryo-trapped in the inlet at −120 °C during pyrolysis and the inlet was heated at a rate of 5 °C/s up to 400 °C at the start of the chromatographic run. The GC inlet was operated in “solvent vent” mode with a vent flow of 100 mL/min and a purge flow of 50 mL/min at 0.01 min. The vent flow ensures consistent pyrolytic conditions, and the purge flow can be adjusted as the split ratio for the chromatographic run. The GC chromatographic conditions were as follows: GC column: 15 m × 0.25 mm × 0.25 μm RXI5-HT (Restek); flow 1.2 mL/min; oven ramp 40 °C (3 min hold) 15 °C/min to 400 °C (2 min hold) operated in constant flow mode. The mass spectrometer was operated at 70 eV with a GC transfer line temperature of 330 °C, an ion chamber (source) temperature of 250 °C, an *m*/*z* range of 10 to 800, and a recording interval of 0.40 s. Samples were placed in a previously flame-cleaned (hand-held butane torch) quartz tube 25 mm long (open both ends) with a small piece of quartz filter (PALL Life Sciences) placed 17 mm from the top to hold the sample.

NIST Automated Mass Spectral Deconvolution and Identification, AMDIS,^30^ was used to calculate RI and extract pyrolyzate peak spectra. AMDIS was developed to analyze GC–MS data of mixtures and even though the JEOL mass spectrometer is a high-resolution instrument, data processing through AMDIS renders the resulting spectra unit resolution. An annotated pyrolysis mass spectral library was made, and an interactive search application was implemented using R^31^ and Shiny.^32^ This application calls AMDIS in the background, utilizes the R package mssearchR^33^ for spectral searching, filters the hitlists, and calculates a final polymer score.

Combined mass spectra of the pyrogram were generated, and cosine similarity (to mimic common current pyrolysis search methods) was used for comparison with NIST PPS. JEOL msAxel software was used to create the combined mass spectra (average over the entire pyrogram with background subtraction in an area with no pyrolyzate peaks), and the R package Co-Operation^34^ was used for the calculation of cosine similarity (see Supporting Information S7).

Since electron ionization (EI) spectra are highly reproducible, any instrumental setup that generates whole-spectrum EI mass spectra (not tandem mass spectra or selected ion monitoring) can be used. NIST PPS can be used if the pyrolysis or any pretreatment steps happen prior to or in-line with the GC/MS.

### 2.2 Development of the Pyrolysis Library

The confident identification of polymers by pyrolysis (Figure 1) requires reproducibility of pyrolysis which can be challenging but is achievable with careful control of experimental parameters and data analysis. Interferences and pyrolyzates common to multiple polymers are frequent, particularly when mixtures or samples with complex matrices are analyzed. Table S2 shows some common pyrolyzates and the polymer(s) they come from as well as retention indices showing close elution of multiple pyrolyzates. Pyrolytic reactions between polymers, additives, or the sample matrix are possible. The copyrolysis of PET and PVC^35^ is one simple example of pyrolytic reactions possible with a complex sample as will be discussed below. In addition, gas chromatography itself has its own limitations in separating closely related compounds, higher molecular weight (>≈800 amu) compounds such as oligomers, or compounds with low volatility. Asymmetric chromatographic peak shapes of highly polar pyrolyzates, such as acids, also present challenges. A ramped pyrolysis method was used due to its flexibility in avoiding pyrolysis at a temperature above the optimal for a diverse set of polymers. A high temperature GC column was utilized to enable the elution of the largest (and most unique) possible pyrolyzates as well as additives.

**Figure 1:**
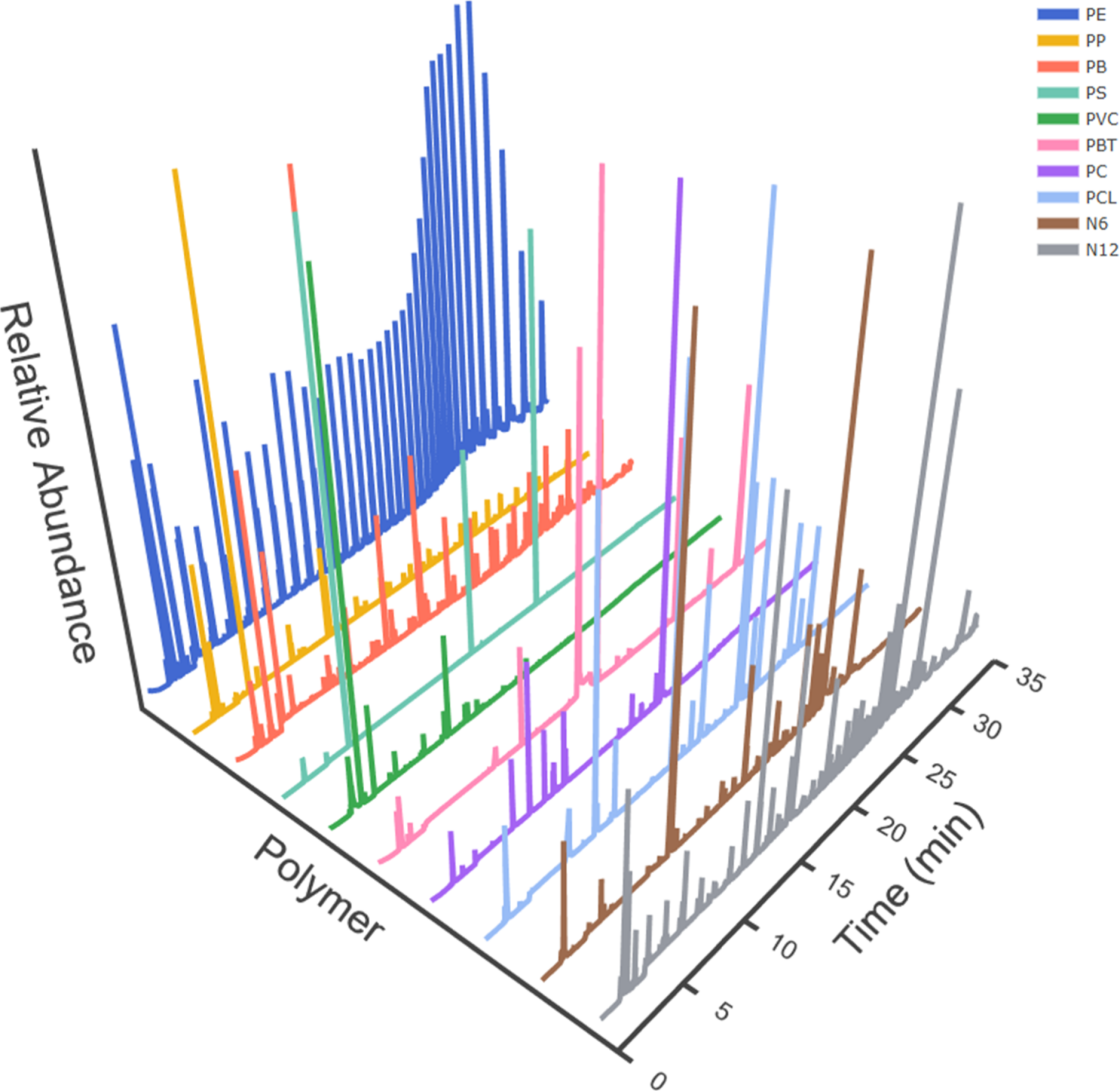
Pyrograms of various polymers demonstrating the diversity of pyrolyzates.

The automated identification of pyrolyzates depends on robust chromatographic matching, which is readily done by linear RI. Throughout this work, RI calculations were based on the ramped pyrolysis method (thermal desorption) of the C7–C40 *n*-alkane standard. Thus, RI values <700 and >4000 are not generally accurate, and pyrolyzates eluting in this realm are omitted if sufficient other pyrolyzates are present. Our aim was to have a repeatable scheme that is robust to changes in experimental conditions (pyrolytic and chromatographic) and sample composition. No open-source py–GC/MS search software and mass spectral library using a multitude of marker compounds for each polymer is known to us.

The pyrolyzate marker compound library was created from the pyrolysis of individual polymer samples. The raw data was processed with AMDIS to extract mass spectra and calculate the RI. Each pyrolyzate marker compound library entry was annotated with structure, formula, molecular weight, exact mass, CAS number (if available), RI, and the polymer(s) from which it arises. The identity of each pyrolyzate was determined either by a match to NIST EI mass spectral main library,^36^ literature search, or rationalization based on pyrolysis principles.^37^ Specific isomers were not determined for diastereomers. There were several pyrolyzates that did not have high confidence in the exact identity, and for which only the molecular formula is given. Catalytic pyrolysis can give rise to different pyrolyzates which would need to be in the library for proper identification of the polymers under catalytic conditions (intentional or from matrix components).

The selection of which pyrolyzates to include as marker compounds for a polymer is of great importance. If there is only one or a few marker compounds, then there is a greater risk of coelution, pyrolyzates common to multiple polymers, or matrix interferences precluding the accurate identification of the polymer. Library pyrolyzates were selected manually (roughly based on the 5% abundance of the largest chromatographic peak) and were chromatographically well resolved. No special consideration was given to asymmetric peaks, but some thought was given to excluding pyrolyzates containing end groups (especially important for low molecular mass polymers). For example, the markers for polyethylene (PE) were defined as alkanes and alkenes up to C23 and some alkadienes, partly because above C23 chromatographic resolution is less likely (depending on the exact experimental conditions—see Figure S5), and the alkadienes are not necessarily present in appreciable amounts especially in the pyrolysis of low-molecular mass PE.

### 2.3 Data Analysis Workflow

A data processing scheme (Figure 2) was developed to identify chromatographic peaks (pyrolyzates) and systematically score the corresponding parent polymers. The algorithm proceeds through the following steps: RI calibration, mass spectral extraction and RI calculation, library searching, RI penalization, unique pyrolyzate hitlist generation, and polymer attribution.

**Figure 2:**
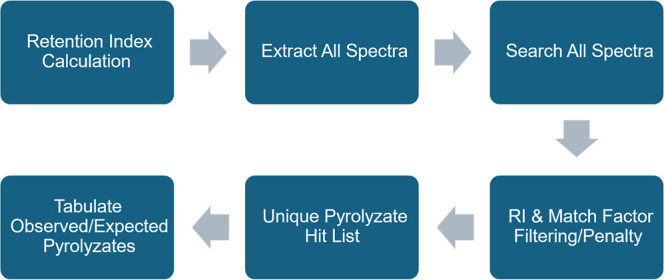
Schematic workflow of NIST PPS.

After RI calibration with the appropriate calibration data, RI determination and spectral extraction of the sample data, then a comprehensive library search is performed for all detected chromatographic peaks. Candidate matches with a match factor (MF) below the selected threshold are discarded. A modified MF (modMF) is calculated by applying an RI penalty to the MF based on the difference in sample RI compared to the library and a selected penalty rate.

1 $$modMF=MF-{RI}_{penalty}$$

where

$${RI}_{penalty}=penalty\;rate\times \frac{\left|\Delta RI-{RI}_{tolerance}\right|}{{RI}_{tolerance}}$$

The default penalty rate was set to 50, and the RI tolerance was defined as RI/100, while the default MF cutoff was set to 700. Hits below the established modMF cutoff are excluded, and the resulting candidate lists for each pyrolyzate are sorted in descending order by modMF. Then, to resolve redundant assignments, the top-ranked candidate for each pyrolyzate is isolated to form a primary subset. In instances where a specific library compound appears as the top hit for multiple pyrolyzates, the assignment with the highest modMF is retained as the putative correct identification. These primary hits are then removed from the lower-ranking (“secondary”) candidate hitlists of all other pyrolyzates. The remaining secondary candidates across all hitlists are then evaluated for redundancies; if any library compound appears multiple times, only the instances corresponding to the highest modMF are retained. Following this pruning procedure, a given chromatographic peak may be represented by zero, one, or multiple candidate hits. Crucially, each library compound is uniquely assigned to a single peak within the pyrogram.

To determine the final attribution score for each parent polymer, the frequency of positive pyrolyzate assignments is quantified. The final score is computed utilizing the following formula

$${FS}_{pol}=\left(\frac{N_{obs}}{N_{\exp}}\right)ave\left(modMF\right)$$

where: FS_pol_ represents the final score for the parent polymer, *N*_obs_ is the number of experimentally attributed pyrolyzates from a specific polymer, *N*_exp_ is the total number of pyrolyzates for that polymer present in the reference library, and ave­(modMF) is the average of all modMF of pyrolyzates attributable to that polymer. A comprehensive step-by-step mathematical representation of this calculation is detailed in the Supporting Information S4. The final score is a type of similarity metric and as with any library search outputting a similarity metric the criteria for defining a “good” match or differentiating between two matches can vary on the specifics of the analysis parameters as well as the sample and is left to the user. No uncertainty value is attached to the final score as there is no consensus on how to determine the uncertainty of a qualitative measurement. The uncertainty for qualitative analysis is often expressed as a probability.^38^ False positive and negative rates would depend on specific parameter choices in the app. These rates could be affected by matrix components obscuring the pyrolyzate peaks or by changes in the pyrolytic behavior such as can happen with catalytic pyrolysis.

## 3 Results and Discussion

### 3.1 Search App

This scheme was developed into an R-Shiny search app (NIST PPS). NIST PPS is self-contained requiring no installation of other software because it has all associated dependencies (AMDIS, R and required R packages) bundled with it. Library expansion is ongoing. As of this publication, it has 168 pyrolyzates from 13 common polymers (see Supporting Information S1 for specifics on the polymers). The app has a user interface that allows loading of a file for RI calibration, automatic generation of the RI calibration file, loading of the sample file, and automatic processing of the pyrolysis data all with minimal user input required. NIST PPS is currently demonstrated to work with *.D and *.CDF files (the only file types available in house for testing). The app currently includes user inputs for modified match factor cutoff, signal-to-noise, and RI penalty rate (see Supporting Information S8).

The results file from AMDIS (*.ELU) is automatically read into the app and searched with the R package “mssearchr” (both during RI calibration as well as pyrolysis search). The results of the search are then displayed on the screen and written to *.csv files (individual files for pyrolyzates and polymer results). The user can manually inspect individual pyrolyzate spectra and compare them to the library in a head-to-tail plot.

NIST PPS does have some potential drawbacks and limitations. Since the library was created with a ramped pyrolysis method, the final polymer scores could be suppressed, or another polymer could be incorrectly attributed, if analyzing flash pyrolysis data. This is due to the potential for more breakdown products at elevated temperatures which could be common to other polymers. The extent of this has not been extensively tested. The RI calibration within the app is done automatically, meaning that, if that process failed, one would need to manually create the calibration file to get proper results (see Supporting Information S3). Each pyrolyzate is not uniquely identified, but all library hits with modified match factors over the cutoff are considered. Thus, a chromatographic peak could be attributed to an incorrect pyrolyzate, inflating the final polymer score for an incorrect polymer. Also, the polymer score for an incorrect polymer will be inflated if a pyrolyzate is common to multiple polymers. These issues are mitigated due to the use of many pyrolyzates as marker compounds for any individual polymer. Finally, since all library RI values were calculated on a semistandard nonpolar column (5% phenyl type, e.g. DB-5 or RXI-5 column), other column phases will have deviations in the RI. Differences of GC parameters such as column phase ratio, gas flow rate, or oven heating rates could also create unacceptable RI differences degrading the pyrolyzate matches, and thus, the final polymer score.

### 3.2 Search App Performance

To understand the performance and potential of this analysis approach and the NIST PPS app for polymer identification by py–GC/MS, experiments were performed focusing on differences in pyrolysis conditions, polymer mixtures, polyethylene blended with a complex organic matrix, and a copolymer. The combined mass spectra were created for all query samples and selected library entries and the cosine similarity between them was calculated to mimic a commonly used pyrolysis search method.

#### 3.2.1 PET at Various Pyrolysis Conditions

Since the pyrolytic degradation pathways of a specific polymer are dependent on the temperature,^39^ PET was pyrolyzed via flash pyrolysis at 450 °C, 500 °C, and 650 °C to test how well NIST PPS would perform with different pyrolysis parameters (Figure 3). The cosine similarity of the combined mass spectra of the pyrograms, as is done in combined mass spectral based searching, is calculated as well. Table 1 summarizes the final polymer scores obtained by NIST PPS in comparison to a combined mass spectrum search method. The NIST PPS final polymer score for PET at 450 °C was 722 and 7 of 8 pyrolyzates were found (abbreviated 722; (7/8)), at 500 °C a score of 710; (7/8), and at 650 °C a score of 718; (7/8) (maximum score of 999; (8/8) for PET). Of note, the RI calibration for these runs was less than ideal, as no calibration peaks corresponding to *n*-alkanes less than C9 were detected (due to evaporation of the lower *n*-alkanes before analysis). This means the calculated RI for benzene (library RI 652) was inaccurate and resulted in a low modMF, which was below the default match factor cutoff of 700. This ultimately caused NIST PPS to omit benzene, one of the markers for PET, from the hitlist. The score for the ramped pyrolysis data used to generate the library, by definition, is 999; (8/8). The cosine similarity (maximum similarity of 1 for identical data) of the combined MS was 0.747 between ramped and flash pyrolysis at 450 °C, 0.987 between ramped and flash at 500 °C, and 0.960 between ramped and flash at 650 °C. The combined MS search method showed a drop off of the cosine similarity with flash pyrolysis at 450 °C (due to incomplete pyrolysis at that low temperature) were with NIST PPS there was minimal difference in final score between the different flash pyrolysis temperatures. Performing flash pyrolysis at an elevated temperature also risks producing more pyrolyzates common to other polymers. Flash pyrolysis of PET at 650 °C also had a score of 628; (4/5) for PVC, while PS and PBT had even lower scores, flash pyrolysis at 450 °C also had a score of 93; (1/9) for PBT, and flash pyrolysis at 500 °C also had a score of 204; (1/4) for PS. Of particular interest, the similar polymers, PET and PBT, are difficult to distinguish by a combined MS method (cosine similarity of 0.747 vs 0.675 at 450 °C, 0.987 vs 0.823 at 500 °C, and 0.960 vs 0.963 at 650 °C) whereas NIST PPS readily distinguished the two at all flash pyrolysis temperatures.

**Figure 3:**
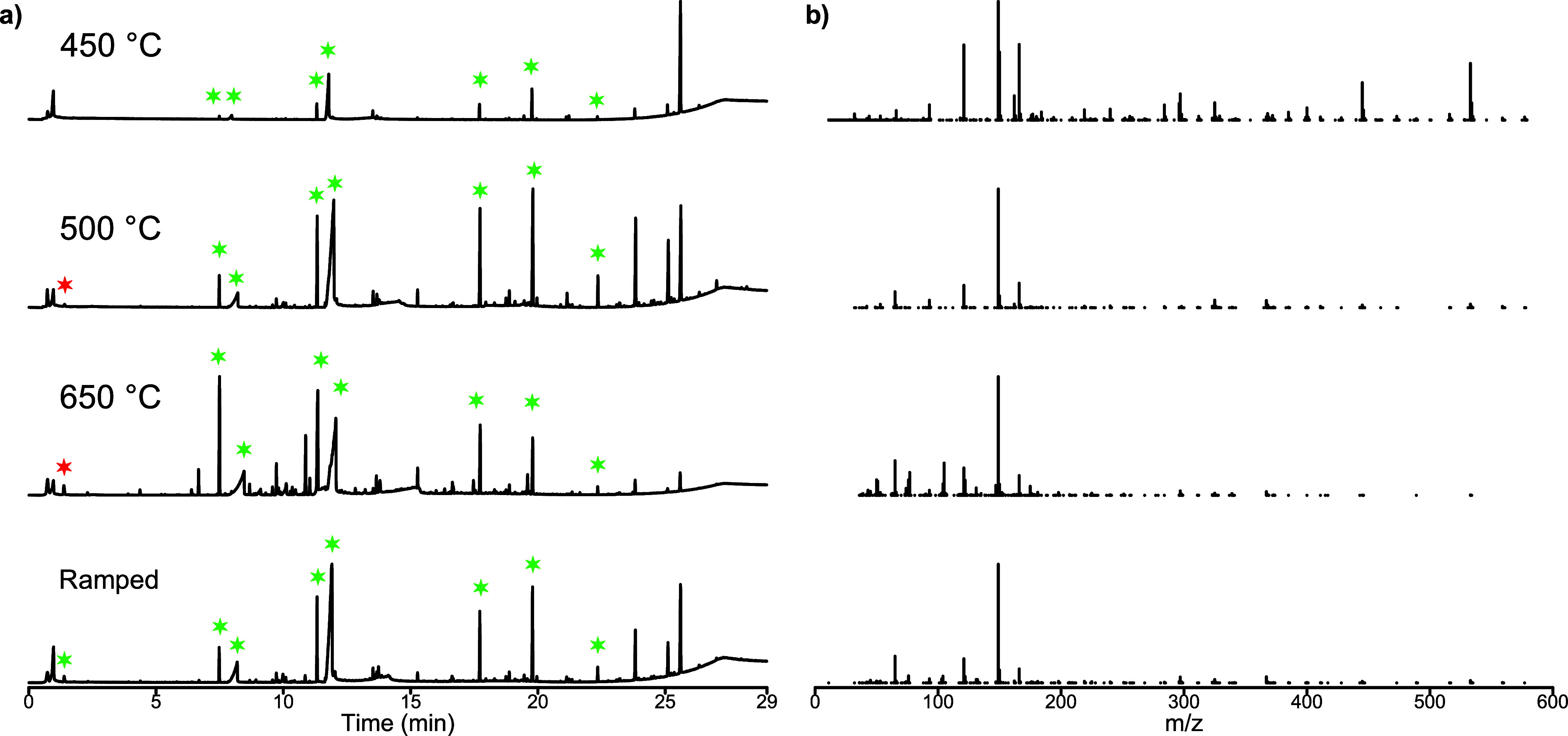
(a) Pyrograms and (b) combined MS of PET flash pyrolysis at different isothermal pyrolysis temperatures and for ramped pyrolysis. Green stars are marker compounds identified and red stars are marker compounds not identified (due to RI mismatch). Combined MS of flash pyrolysis at different temperatures show significant differences.

**Table 1:** Comparison of Results from NIST PPS and Combined MS for Flash Pyrolysis of PET at Different Temperatures^a^

	comparison to library (ramped pyrolysis)							
	PET		PVC		PBT		PS	
flash pyrolysis PET (°C)	NIST PPS score	combined MS cosine similarity	NIST PPS score	combined MS cosine similarity	NIST PPS score	combined MS cosine similarity	NIST PPS score	combined MS cosine similarity
450	722; (7/8)	0.747	-	-	93; (1/9)	0.675	-	-
500	710; (7/8)	0.987	-	-	164; (2/9)	0.823	204; (1/4)	0.018
650	718; (7/8)	0.960	628; (4/5)	0.150	166; (2/9)	0.963	408; (2/4)	0.155

a RI calibration difficulties limited matches for pyrolyzate RI < 900.

#### 3.2.2 Polyolefin Discrimination

Discriminating polyolefins is challenging because of the large number of pyrolyzates and common or similar pyrolyzates from the different polyolefins. Figure 4 shows the pyrograms of PE, PP, and PB and a mixture of the three polyolefins as well as the combined mass spectrum for each, and Table 2 summarizes the NIST PPS and combined mass spectrum search results.

**Figure 4:**
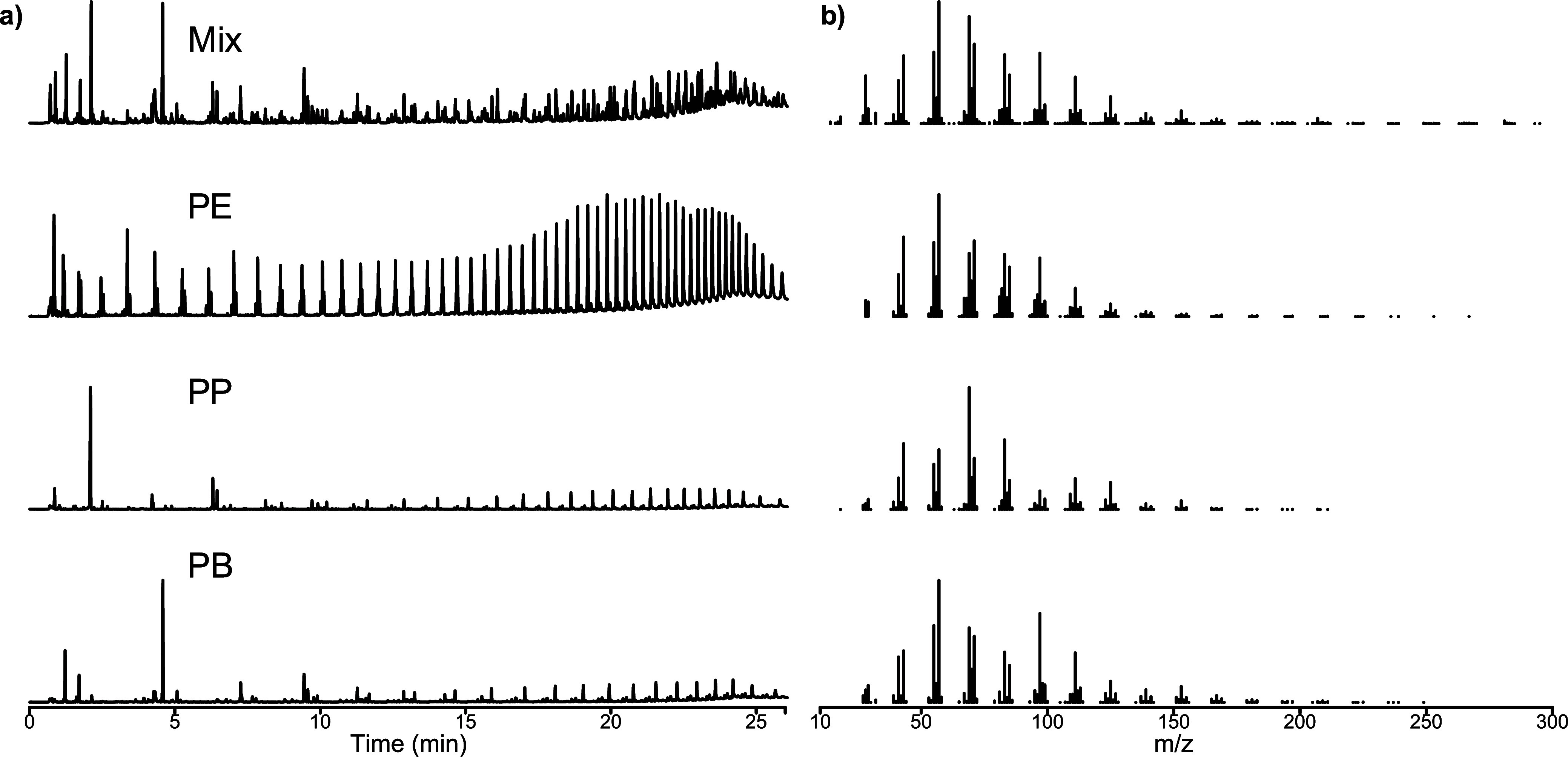
(a) Pyrograms and (b) combined MS of polyolefin mixture (Mix), PE, isotactic PP (PP), and isotactic PB (PB). Note marker compounds are too numerous to demarcate clearly on the pyrograms.

**Table 2:** Comparison of Results from NIST PPS and Combined MS Methods for a Mixture of Polyolefins^a^

	PE		PP		PB	
sample	NIST PPS score	combined MS cosine similarity	NIST PPS score	combined MS cosine similarity	NIST PPS score	combined MS cosine similarity
PE/PP/PB	807; (44/48)	0.964	913; (22/23)	0.921	914; (28/28)	0.696
PE	920; (47/48)	1	196; (6/23)	0.869	86; (3/28)	0.951
PP	208; (13/48)	0.869	891; (22/23)	1	382; (14/28)	0.855
PB	250; (16/48)	0.951	620; (19/23)	0.855	924; (28/28)	1

a This data is included with the app as demo data.

The cosine similarity between the combined mass spectrum of the sample to the standards was calculated: Mix/PE—0.964, Mix/PB—0.969, Mix/PP—0.921. The cosine similarities between the combined mass spectra for the reference PE/PP/PB were also calculated: PE/PP—0.869, PE/PB—0.951, and PP/PB—0.855. The high similarities between the individual polyolefins indicate that it is difficult to differentiate the polyolefins using this technique. Using NIST PPS the final polymer scores are 914; (28/28) for iso-PB, 913; (22/23) for iso-PP, and 807; (44/48) for PE. Of note, as currently implemented NIST PPS, is very sensitive to deviations in RI especially for early eluting compounds. This is partly due to the RI tolerance being specified as RI/100 and partly due to the calibration of RI from a C7–C40 *n*-alkane mix making RI values less than 700 unreliable. This can be mitigated by changing the match factor cutoff; better handling of the RI is an active area of investigation. In this instance the depressed score for PE in the mixture is partly due to the RI mismatch penalty for 1-hexene (library RI of 621), giving rise to a match factor penalty of 208, resulting in a modified match factor of 730. Also, since this is a complex mixture successful deconvolution of overlapping peaks is difficult resulting in some markers not being detected. NIST PPS scores for the pure polymers were not the maximum (e.g. PE score is 920; 47/48 vs maximum of 999; 48/48) due to experimental variation in the RI and mass spectra, whereas the summed MS used for cosine similarity were the maximum of 1 because they were only generated and used for this comparison. There are very few common pyrolyzates between the polyolefins, but many are similar enough in retention time and mass spectral similarity to be attributable as a pyrolyzate from multiple polyolefins.

#### 3.2.3 PET and PVC Discrimination

The pyrolysis of a mixture of PET and PVC is also difficult to deconvolute. The pyrograms and combined mass spectra are shown in Figure 5 and Table 3 summarizes the NIST PPS and combined mass spectrum search results. Since the pyrogram of PVC is dominated by benzene (which is common with PET), and PET produces many pyrolyzates, it is difficult to detect PVC in the mixture via the combined mass spectra method. Also of note, the pyrolysis of PVC involves the elimination of HCl which then reacts with the PET to create byproducts in the copyrolysis of the mixture. Cosine similarity between the individual combined mass spectra was calculated: mix/PET—0.983, mix/PVC—0.264, and PET/PVC—0.119. Using a combined MS method is susceptible to missing PVC due to lower abundance pyrolyzates being masked by more abundant pyrolyzates and reaction products. Using NIST PPS the final polymer scores are 949; (8/8) for PET and 936; (5/5) for PVC. NIST PPS scores for the pure polymers were not the maximum due to experimental variation, whereas the summed MS used for cosine similarity were the maximum because they were only generated and used for this comparison.

**Figure 5:**
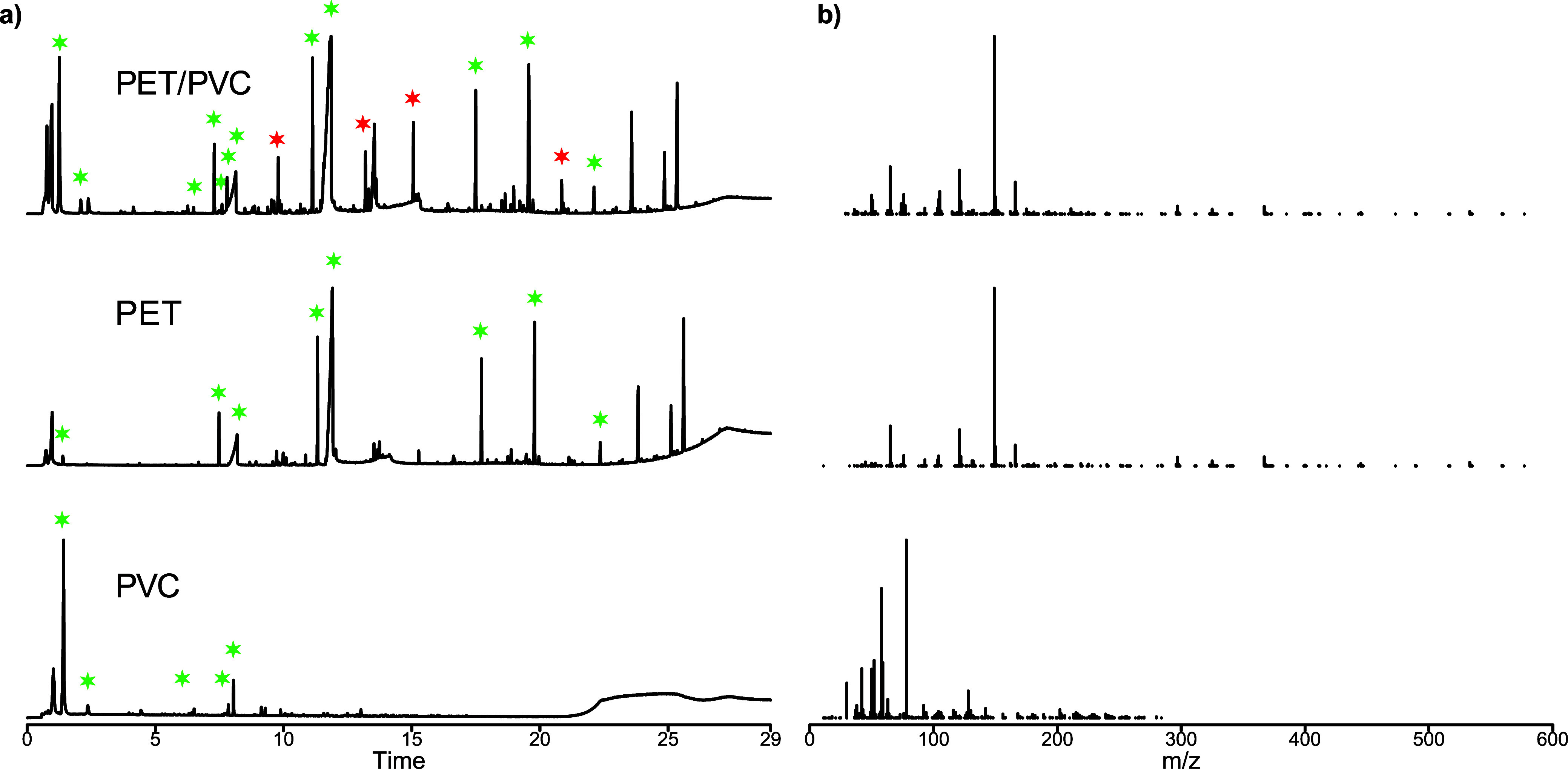
(a) Pyrograms and (b) combined MS of PET/PVC mixture, PET and PVC. Green stars are marker compounds, and the red stars are reaction products between PET and PVC.

**Table 3:** Comparison of Results from NIST PPS and Combined Mass Spectrum Methods for a Mixture of PET and PVC

	PET		PVC	
sample	NIST PPS score	combined MS cosine similarity	NIST PPS score	combined MS cosine similarity
PET/PVC mix	949; (8/8)	0.983	936; (5/5)	0.264
PET	838; (8/8)	1	188; (1/5)	0.119
PVC	103; (1/8)	0.119	916; (5/5)	1

#### 3.2.4 Copolymer Analysis

A sample of styrene *n*-butyl methacrylate copolymer (SBMA) was run to see how robust this method is for copolymers. The pyrograms and combined mass spectra are shown in Figure 6 and Table 4 summarizes the NIST PPS and combined mass spectrum search results. The type of copolymer (random or block) and the ratios of the monomers are expected to have an impact on the pyrolysis results. The sample was a random copolymer with no specified ratio of the monomers. Seven pyrolyzates were chosen as markers for SBMA with one common to poly *n*-butyl methacrylate (PBMA), three common to PS, and three unique to SBMA (presumed to be styrene/butyl methacrylate mixed trimers). With the common pyrolyzates, the NIST PPS score was 946; (4/4) for PS and 949; (1/1) for PBMA. This shows the importance of inspecting the whole hitlist as the copolymer could easily be mis-identified as either homopolymer. Also, a mixture of either homopolymer with the copolymer would be impossible to deconvolute. The cosine similarities for the combined mass spectra are 0.618 for SBMA/PBMA, 0.711 for SBMA/PS, and 0.024 for PBMA/PS. It is expected that pyrolysis of block copolymers (especially with larger block sizes) would approach that of a mixture of the two pure polymers since the amount of the mixed *n*-mer would approach the limit of detection in comparison to the pyrolyzates of the pure polymers. Similarly, as the ratio of one monomer approached zero the ability to detect the mixed *n*-mer goes to zero and a successful identification of the copolymer is expected to decrease. The analysis of this copolymer samples shows promise for this approach to analyze copolymers; however, significant additional work would be needed to determine robustness for other copolymers due to the points identified above. NIST PPS scores for the pure polymers were not the maximum due to experimental variation, whereas the summed MS used for cosine similarity were the maximum because they were only generated and used for this comparison.

**Figure 6:**
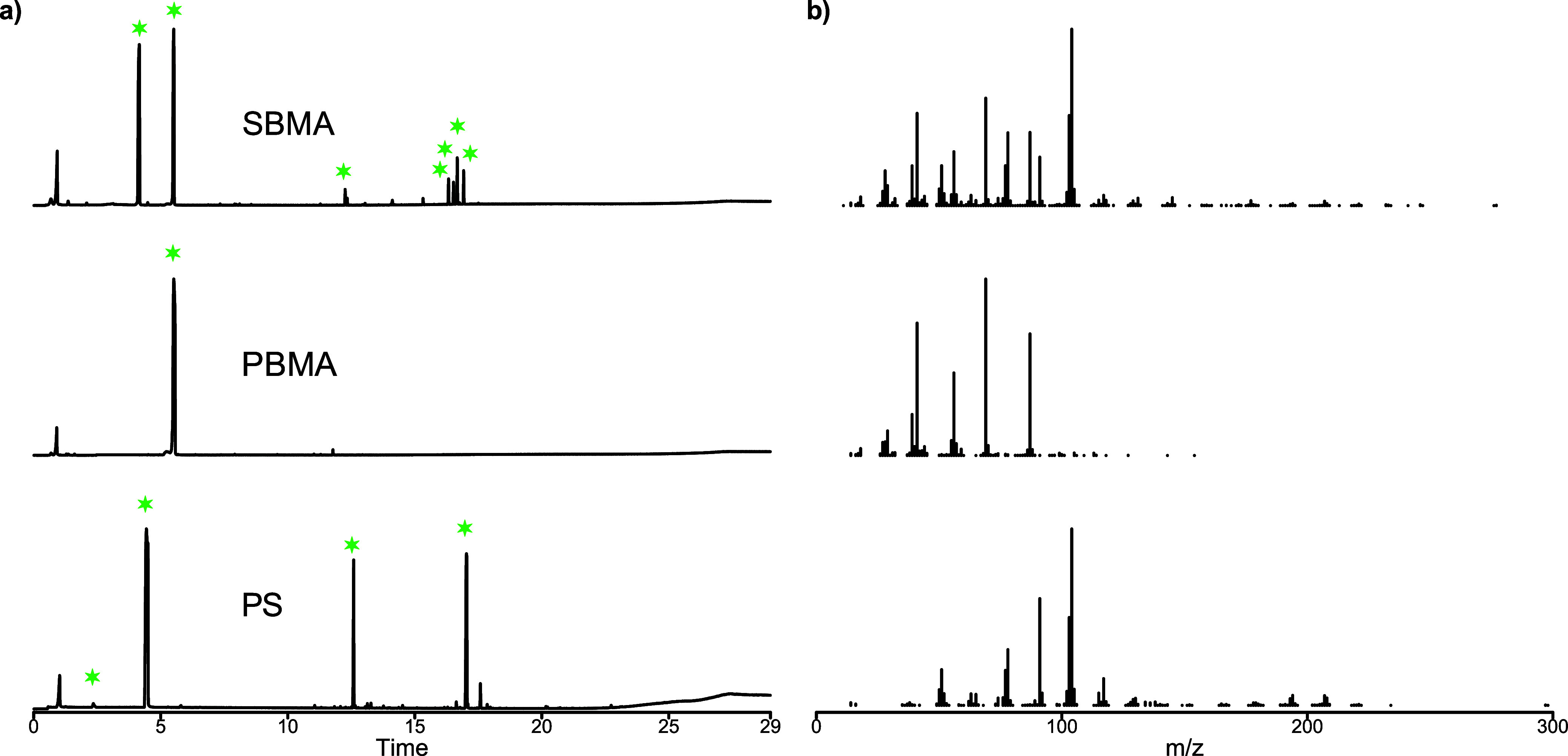
(a) Pyrograms and (b) combined MS of SBMA, PBMA, and PS. Green stars designate marker compounds.

**Table 4:** Comparison of Results from NIST PPS and Combined Mass Spectrum Methods for a Copolymer and the Individual Homopolymers

	SBMA		PBMA		PS	
sample	NIST PPS score	combined MS cosine similarity	NIST PPS score	combined MS cosine similarity	NIST PPS score	combined MS cosine similarity
SBMA	968; (7/7)	1	949; (1/1)	0.618	946; (4/4)	0.711
PBMA	139; (1/7)	0.618	975; (1/1)	1	0; (0/4)	0.024
PS	412; (3/7)	0.711	0; (0/1)	0.024	961; (4/4)	1

#### 3.2.5 Discrimination of Polyolefins in a Complex Matrix

As previously mentioned, the misidentification/over quantitation of PE in biological samples is a concern^21^ due to the fat and protein content. The pyrolysis of proteins generates an elevated chromatographic baseline which makes it difficult to detect PE, and flash pyrolysis of fats can generate common pyrolyzates to PE. The flash pyrolysis of fats generating pyrolyzates common to PE could be an artifact of specific experimental parameters as attempts to replicate this issue with our system have had minimal success (see Supporting Information S5). One strategy to mitigate this is to remove the matrix prior to pyrolysis. A version of this was tested to see if it would interfere with the performance of the app. A consumer protein powder drink mix was used as a surrogate for a complex biological matrix and pyrolyzed, resulting in a complex pyrogram that is difficult to deconvolute. Using the combined MS method, the unadulterated protein powder has a cosine similarity of 0.513 with PE while with NIST PPS the PE score is 0; 0/48. The high cosine similarity score is due to the complex pyrogram and pyrolyzates having somewhat similar spectra to PE. An approximate 7:1 mixture by mass of protein powder to PE was prepared and pyrolyzed. The pyrograms and combined mass spectra are shown in Figure 7 and Table 5 summarizes the NIST PPS and combined mass spectrum search results. The background due to the protein powder was significant, and analysis with NIST PPS resulted in a score of 493; (29/48) for PE vs 0.470 for the combined mass spectrum. This final score is relatively low, as only 29 of 48 markers for PE were detected due to the high background. Upon inspection of the chromatogram, the user could decide whether such a score would be acceptable for identifying PE. An online pretreatment THM step was performed before the pyrolysis to determine whether it would help eliminate matrix interferences of PE. This THM step was carried out by adding TMAH to the sample mixture in the pyrolysis tube and heating it at a temperature lower than pyrolysis. Analysis of the THM data of the protein powder/PE mixture detected no PE pyrolyzates indicating no PE breakdown occurring during this step (not shown). The residue in the pyrolysis tube was then pyrolyzed with the normal ramped pyrolysis method. Analysis of the pyrolysis data indicated continued elevated background, but PE was detected with an improved score of 826; (45/48). On the other hand, the combined MS method performed approximately the same (0.476 vs 0.470) as without pretreatment. Optimization of the THM pretreatment conditions could possibly improve this result perhaps even to a mixture of lower PE concentration that is biologically plausible. Obviously, if one uses a pyrolytic method in which the pyrolysis of fats generates pyrolyzates common to PE, NIST PPS will not eliminate misidentification, but we demonstrate that a harsh THM pretreatment step does not interfere with using this data analysis method.

**Figure 7:**
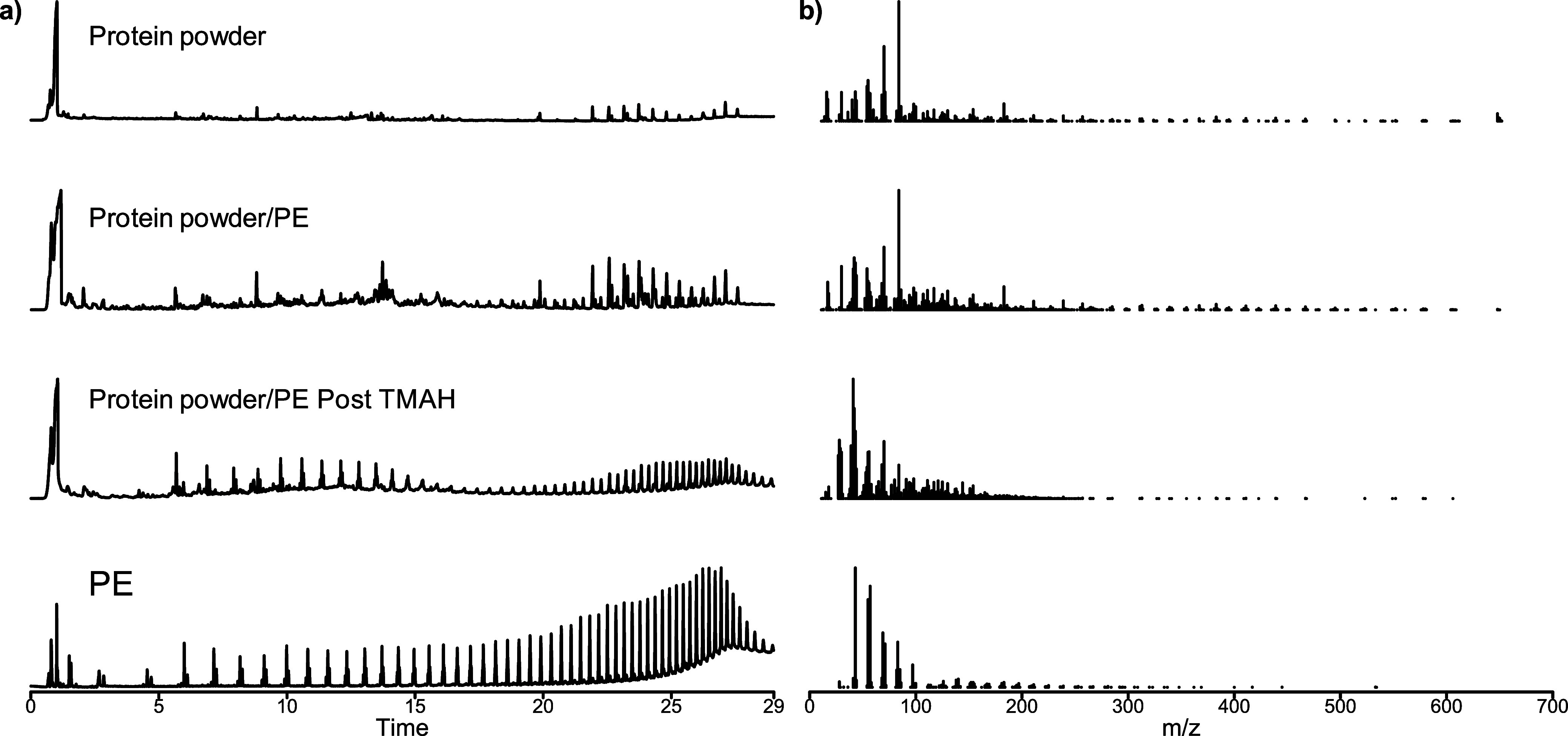
(a) Pyrograms and (b) combined MS of protein powder, protein powder/PE mixture, pyrolysis of post THM material, and PE. Note marker compounds are too numerous to demarcate in the figure.

**Table 5:** Comparison of Results from NIST PPS and Combined Mass Spectrum Methods for Polyethylene in a Complex Matrix

	PE	
sample	NIST PPS score	combined MS cosine similarity
protein powder	0; (0/48)	0.513
protein powder & PE	493; (29/48)	0.470
post TMAH treatment	826; (45/48)	0.476

### 3.3 Implications and Future Directions

This work demonstrates an increase in the identification confidence of polymers, polymer mixtures, and even polymers blended with complex matrices using NIST PPS, a purposely designed pyrolysis library and search app. The library and search app are freely available as a self-contained download (no other software needed) at https://chemdata.nist.gov/dokuwiki/doku.php?id=chemdata:polymersearch.

While the results presented here indicate the promise of an approach based on the automated searching of a pyrolyzate reference library and consideration of multiple pyrolyzates marker compounds, significant future work is needed to fully understand the performance and limitations as the effort so far has encompassed a limited number of materials and experimental conditions. Future efforts are needed related to expanding the library (including catalytic pyrolysis), investigating the impact of experimental conditions, and in refinement of the search app.

First, additional polymers need to be added to the library. In addition to expansion of types of polymers, investigation of differences in grades (e.g., molecular weight and additives) would be fruitful for understanding robustness. Prioritizing which polymers and copolymers to include next is an ongoing process. In principle, a natural continuation of this process will be adding real-world materials with high levels of additives, provided the quality control of the library is acceptable, as these materials would be an excellent test for the library and search app performance.

Second, an expanded investigation of the impact of experimental conditions is needed. For example, investigating the changes in pyrolyzates for different pyrolysis temperature ramp rates is of interest. Testing different thermochemolytic reagents may be a fruitful line of inquiry. It is possible that improvements in speed and/or sample loading can be made without degrading the chromatographic separation to an undesirable level by using low-pressure gas chromatography.

Third, continued refinements of the search app are desired. Further testing on how to best treat RI differences in pyrolyzates is an area of interest. This is especially important for areas where RI calibration compounds are absent or where chromatographic resolution is minimal (close to the void volume or elevated temperature).

This work attempts to move forward the state of qualitative identification. An important part of this analysis should include quantitation and can only be addressed after reasonably accurate qualitative identification has been made and is beyond the scope of this work.

While the results, approach, and future directions identified here indicate promise in improving polymer identification via py–GC/MS, significant challenges remain for the rapid and robust determination of the composition and concentration of polymers in complex samples.

## Supplementary Material



## Notes

List of materials used, partial list of pyrolyzates in the library, file paths and description of background files used by NIST PPS, example of hitlist filtering, some specifics on PE pyrolysis and potential interferences, and known issues with running NSIT-PPS (PDF)

## Abbreviations

NIST PPS: NIST polymer pyrolysis search
PE: polyethylene
PP: polypropylene
PB: polybutylene
PS: polystyrene
PVC: polyvinyl chloride
PET: polyethylene terephthalate
PBT: polybutylene terephthalate
PC: polycarbonate
PCL: polycaprolactone
N6: nylon-6
N12: nylon-12
PBMA: poly *n*-butyl methacrylate
SBMA: styrene–butylene methacrylate copolymer
MS: mass spectrum/mass spectrometry
py–GC/MS: pyrolysis gas chromatography mass spectrometry
TMAH: tetramethylammonium hydroxide
