# Supplementary material for: NIST Polymer Pyrolysis Search: A New Pyrolysis GC–MS Search Program and Mass Spectral Reference Library
Source: Environ Sci Technol. 2026 Jun 29;60(27):19336–46. doi: 10.1021/acs.est.5c18872 (PMC13374098; doi:10.1021/acs.est.5c18872)
Supplement: Supplementary file 1 [file es5c18872_si_001.pdf]

Supporting Information

NIST Polymer Pyrolysis Search: A new pyrolysis GC-MS search program and mass spectral reference library

Edward P. Erisman*, Yamil Simón-Manso, William E. Wallace

Mass Spectrometry Data Center, Biomolecular Measurement Division, National Institute of Standards and Technology, Gaithersburg, Maryland 20899, United States.

Summary of supplemental information:

Abbreviations	page S1
S1: materials used	page S2
S2: Selected pyrolyzates, RI, and parent polymer	page S2-S3
S3: File formats and locations of the search application	page S3-S4
S4: Example data analysis calculations done manually	page S4-S6
S5: Some complexities of the pyrolysis of polyethylene	page S6-S10
S6: Known issues with the search application	page S10
S7: Cosine similarity of combined mass spectra	page S10
S8: Screen shot of NIST PPS search app	page S11

ABBREVIATIONS

NIST-PPS, NIST polymer pyrolysis search; PE, polyethylene; PP, polypropylene; PB, polybutylene; PS, polystyrene; PVC, polyvinylchloride; PET, polyethylene terephthalate; PBT, polybutylene terephthalate; PBMA, poly n-butyl methacrylate; SBMA, styrene-butylene methacrylate copolymer; MS, mass spectrum/mass spectrometry; py-GC/MS, pyrolysis gas chromatography mass spectrometry; TMAH, tetramethyl ammonium hydroxide.

S1: Tables of materials used to create and test NIST PPS

Material	Abbreviation	Source	Description Catalog # : MW*
Low density Polyethylene	PE	Scientific Polymer Products Inc	042 : 50,000
Isotatic-polypropylene	Iso-PP	Scientific Polymer Products Inc	130 :
Isotatic-poly(1-butene)	Iso-PB	Scientific Polymer Products Inc	128 :
Nylon12	N12	Scientific Polymer Products Inc	044 :
Nylon6	N6	Scientific Polymer Products Inc	034 : 25,000
Polystyrene	PS	Scientific Polymer Products Inc	039A : 260,000
Polycarbonate	PC	Scientific Polymer Products Inc	954 : 36,000
Polycaprolactone	PCL	Scientific Polymer Products Inc	1029 : 25,000
Poly(vinyl chloride)	PVC	Scientific Polymer Products Inc	038 : 275,000
Poly(butylene terephthalate)	PBT	Scientific Polymer Products Inc	962 :
Poly(n-butyl methacrylate)	PBMA	Scientific Polymer Products Inc	111 : 180,000
Styrene/butyl methacrylate copolymer	SBMA	Scientific Polymer Products Inc	595 : 200,000
Poly(ethylene terephthalate)	PET	Goodfellow Advanced Materials	ES30-PD-000132 :
Protein Powder	-	Rise Wellness	Chocolate Almond flavor whey protein

* MW = molecular weight presumed to be (g/mol) as units not reported by the supplier

Mixture	Component 1	Mass fraction	Component 2	Mass fraction	Component 3	Mass fraction	Section
polyolefins	PE	0.26	PP	0.32	PB	0.41	3.2.2
PET/PVC	PET	~ 1	PVC	~ 1	-	-	3.2.3
Protein powder/PE	Protein powder	0.87	PE	0.12	-	-	3.2.5

S2: Table of selected pyrolyzates from the library created for this work to demonstrate the occurrence of pyrolyzates common to multiple polymers (**BOLD**) and the presence of closely eluting (difficult to chromatographically resolve) pyrolyzates (***italics***).

Pyrolyzate	RI	Polymer(s)

2-Methyl-1-pentene	585.8	Iso-PP
1-Hexene	589.3	PE
Benzene	651.8	**PVC; PBT; PET**
1-Heptene	683	**PE; Nylon 12**
Heptane	700	PE; iso-PB
Toluene	759.4	PVC; PS
2-Ethylhexane	760.2	Iso-PB
2-Ethyl-1-hexene	777.5	Iso-PB
1-Octene	784.8	**PE; Nylon 12**
Octane	800	PE
2,4-Dimethyl-1-heptene	831.4	Iso-PP
3-Methyl-5-methyleneheptane	839.4	Iso-PB
5-Hexenenitrile	855	Nylon 6
2,4,6-Trimethyl-1-heptene	884.9	Iso-PP
1-Nonene	887.5	**PE; Nylon 12**
Styrene	892.7	PS
Nonane	900	PE
Phenol	978.6	PC
1,9-Decadiene	979.9	Nylon 12
1-Decene	989.3	**PE; Nylon 12**
Decane	1000	PE
5-Hexenoic acid	***1013.3***	***PCL***
5-Ethylnon-3-ene *	***1014.8***	***Iso-PB***
Indene	1042	PVC

S3: Background files for advanced implementation of NIST PPS:
RI calibration file location:
./NIST_PPS/AMDIS/LIB/Local.cal

RI calibration file format:
Retention_time_1 retention_index_1
Retention_time_2 retention_index_2
…

Result csv files location:
./NIST_PPS/results/

Results csv files:
"polymerlist.csv" – final polymer search result displayed in GUI
"peaksresults.csv"- final filtered hit list for all pyrolyzates

“searchresults.csv”- hit list for all pyrolyzates before filtering but with match factor cutoff applied

Search library location:
./NIST_PPS/libraries/

Library files:
“pyrolysis.MSPEC”- pyrolyzate mass spectral library
“RILIB.MSPEC”- RI calibration library

S4: Example data analysis calculation:
SBMA copolymer (used to generate the library entries so dri =0 for pyrolyzates unique to SBMA)
Relevant data from “searchresults.csv”- unfiltered results
Abbreviations used:

mf: raw modified match factor
dri: delta RI = |sample RI – library RI|
tol: RI tolerance = RI/100
mfp: match factor penalty = 50*(dri – tol)/tol (if dri > tol)
modmf: modified match factor = mf-mfp

**Name**	**mf**	**dri**	**tol**	**mfp**	**modmf**	**ri**	**rt**
Toluene	954.68	9.70	7.50	14.69	939.98	749.7	2.0857
Styrene	923.51	11.30	8.81	14.10	909.41	881.4	4.0666
Styrene	962.94	6.20	8.87	0.00	962.94	886.5	4.1579
n-Butyl methacrylate	977.84	17.10	9.79	37.35	940.49	978.8	5.5096
n-Butyl methacrylate	984.14	16.60	9.79	34.75	949.39	979.3	5.5169
2,4-Diphenyl-1-butene	938.80	1.40	17.16	0.00	938.80	1715.7	12.2599
Docosane	870.38	2.40	21.98	0.00	870.38	2197.6	15.3265
Heneicosane	903.30	97.60	21.98	172.06	731.24	2197.6	15.3265
SBMA trimer 1	994.58	0.00	23.80	0.00	994.58	2380.1	16.3283
SBMA trimer 2	952.72	37.20	23.80	28.15	924.57	2380.1	16.3283
SBMA trimer 2	994.97	0.00	24.17	0.00	994.97	2417.3	16.5242
SBMA trimer 1	943.04	37.20	24.17	26.95	916.09	2417.3	16.5242
2,4,6-Triphenyl-1-hexene	943.45	9.30	24.46	0.00	943.45	2446.2	16.6735
SBMA trimer 3	986.27	0.00	24.95	0.00	986.27	2494.8	16.9241

Note:

Styrene and n-Butyl methacrylate both have high match factors for different peaks. This is due to the AMDIS settings for extracting the spectra, and those peaks are very large and poorly shaped.

The spectra for SMBA trimer 1 and 2 are very similar and elute close together, meaning that without filtering, they both appear as hits for each peak.

Filtering process:

1. Subset the top hit (highest modmf) for each peak from the rest

Top hit:

Name	Mf	dri	tol	mfp	modmf	ri	rt
Toluene	954.68	9.70	7.50	14.69	939.98	749.7	2.0857
Styrene	923.51	11.30	8.81	14.10	909.41	881.4	4.0666
Styrene	962.94	6.20	8.87	0.00	962.94	886.5	4.1579
n-Butyl methacrylate	977.84	17.10	9.79	37.35	940.49	978.8	5.5096
n-Butyl methacrylate	984.14	16.60	9.79	34.75	949.39	979.3	5.5169
2,4-Diphenyl-1-butene	938.80	1.40	17.16	0.00	938.80	1715.7	12.2599
Docosane	870.38	2.40	21.98	0.00	870.38	2197.6	15.3265
SBMA trimer 1	994.58	0.00	23.80	0.00	994.58	2380.1	16.3283
SBMA trimer 2	994.97	0.00	24.17	0.00	994.97	2417.3	16.5242
2,4,6-Triphenyl-1-hexene	943.45	9.30	24.46	0.00	943.45	2446.2	16.6735
SBMA trimer 3	986.27	0.00	24.95	0.00	986.27	2494.8	16.9241

2. If a pyrolyzate appears more than once remove all but the highest modmf

Name	mf	dri	tol	mfp	modmf	ri	rt
Toluene	954.68	9.70	7.50	14.69	939.98	749.7	2.0857
Styrene	962.94	6.20	8.87	0.00	962.94	886.5	4.1579
n-Butyl methacrylate	984.14	16.60	9.79	34.75	949.39	979.3	5.5169
2,4-Diphenyl-1-butene	938.80	1.40	17.16	0.00	938.80	1715.7	12.2599
Docosane	870.38	2.40	21.98	0.00	870.38	2197.6	15.3265
SBMA trimer 1	994.58	0.00	23.80	0.00	994.58	2380.1	16.3283
SBMA trimer 2	994.97	0.00	24.17	0.00	994.97	2417.3	16.5242
2,4,6-Triphenyl-1-hexene	943.45	9.30	24.46	0.00	943.45	2446.2	16.6735
SBMA trimer 3	986.27	0.00	24.95	0.00	986.27	2494.8	16.9241

3. Compare to the “secondary” hits for each peak if any replicates

Name	mf	dri	tol	mfp	modmf	ri	rt
Heneicosane	903.30	97.60	21.98	172.06	731.24	2197.6	15.3265
SBMA trimer 2	952.72	37.20	23.80	28.15	924.57	2380.1	16.3283
SBMA trimer 1	943.04	37.20	24.17	26.95	916.09	2417.3	16.5242

4. Since both SBMA trimer 1 and 2 hits are already a top hit for a different peak, they are discarded.
5. The top hit and secondary hit lists are combined

Final peak results

Name	mf	dri	tol	mfp	modmf	ri	rt
Toluene	954.68	9.70	7.50	14.69	939.98	749.7	2.0857
Styrene	962.94	6.20	8.87	0.00	962.94	886.5	4.1579
n-Butyl methacrylate	984.14	16.60	9.79	34.75	949.39	979.3	5.5169
2,4-Diphenyl-1-butene	938.80	1.40	17.16	0.00	938.80	1715.7	12.2599
Docosane	870.38	2.40	21.98	0.00	870.38	2197.6	15.3265
Heneicosane	903.30	97.60	21.98	172.06	731.24	2197.6	15.3265
SBMA trimer 1	994.58	0.00	23.80	0.00	994.58	2380.1	16.3283
SBMA trimer 2	994.97	0.00	24.17	0.00	994.97	2417.3	16.5242
2,4,6-Triphenyl-1-hexene	943.45	9.30	24.46	0.00	943.45	2446.2	16.6735
SBMA trimer 3	986.27	0.00	24.95	0.00	986.27	2494.8	16.9241

Note: pyrolyzate at 15.3265 min can be attributed to docosane (modmf = 870) or heneicosane (modmf = 731) with matchfactor cutoff of 700. Both are PE pyrolyzates inflating the final score for PE since that peak cannot be both. This is not significant since for PE there are 48 pyrolyzates in the library. The source of this peak is unknown and is likely contamination since it not easily rationalized to be from SBMA.

6. The polymers from which these pyrolyzates arise are then tabulated to get the polymerlist

Abbreviations used:

count: number of pyrolyzates found for a particular polymer
libcount: number of pyrolyzates in the library for a particular polymer
meanmf: mean match factor of all pyrolyzates found for a particular polymer
score = count/libcount*meanmf

polymer	count	librcount	meanmf	score
SBMA	7	7	967.2	967.2
PBMA	1	1	949.39	949.39
PS	4	4	946.29	946.29
PVC	1	5	939.98	188
PE	2	48	800.81	33.37

S5: Pyrolysis of PE:

PE will generally pyrolyze into alkenes, alkanes, and alkadienes (in order of abundance; elution order is alkadiene, alkene, and alkane). These will elute at a very similar time for the same carbon number making it hard to chromatographically distinguish these pyrolyzates. For the method used, these pyrolyzates are not resolved after ≈ C23.

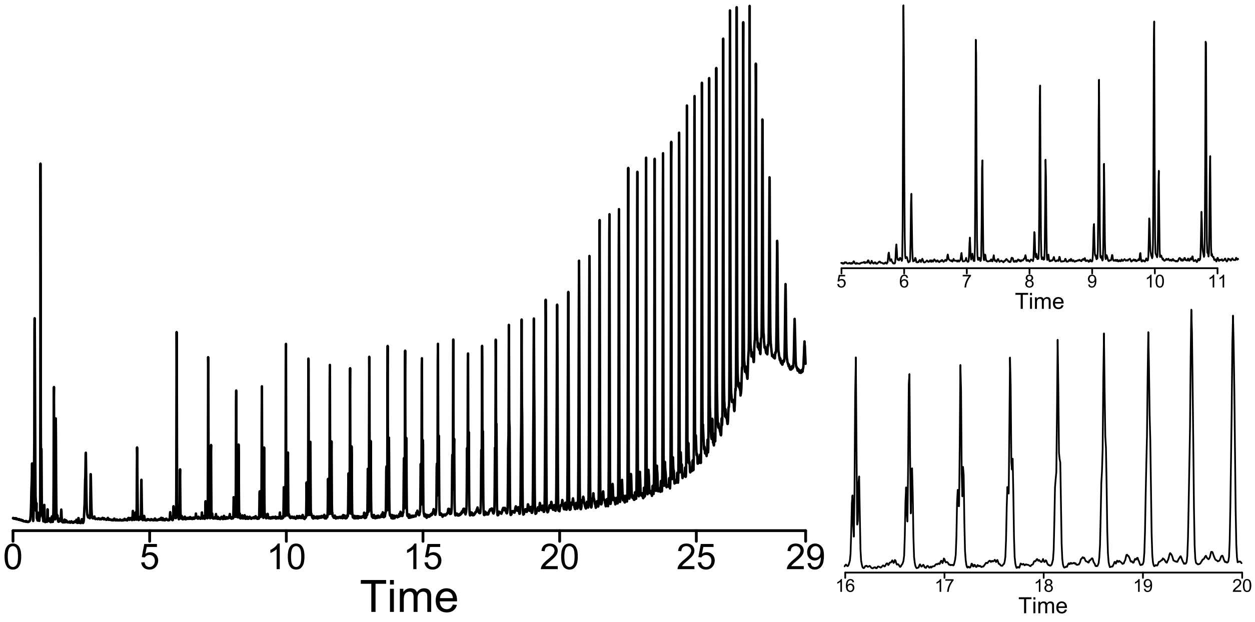

Full pyrogram and zoomed portions of polyethylene (Scientific Polymer Products Inc cat# 042 – approximate MW: 50,000)

The pyrolysis of PE is, to a certain extent, dependent on the molecular weight of the polymer. This is evidenced by comparing the pyrolysis of PE microspheres from Cospheric LLC (part # BLPMS-1.00 500 µm to 600 µm diameter, density adjusted to 1.00 g/mL with titanium dioxide, and colored blue) with the PE from Scientific Polymer Products Inc (above). For the Cospheric LLC sample, which is believed to be a low molecular weight PE, the odd carbon number alkanes (likely from the low MW PE) are visibly different than the pyrolyzates of every carbon number, and the lack of the alkadienes makes the pyrogram

significantly different.

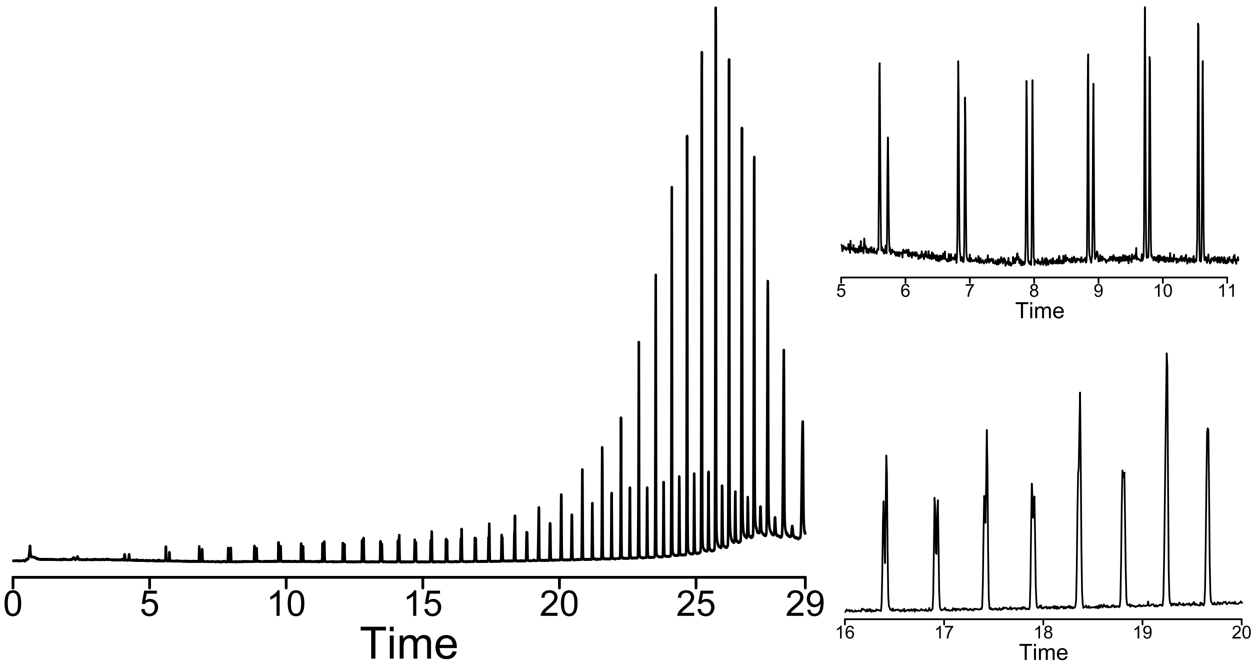

Full pyrogram and zoomed portions of Cospheric microspheres

The pyrolysis of fat has been reported to have common pyrolyzates to PE[1]. These reports utilize the Frontier Laboratories pyrolyzer, employing a flash pyrolysis method and a lower maximum temperature GC oven ramp. We attempted to replicate this interference on our system but were unable to completely replicate it. This is under further investigation.

Glycerol trioleate (GTO) from Scientific Polymer Products Inc (cat# P-154) was used as a surrogate for fats during testing and run by ramp and flash (650 °C) pyrolysis.

(1) Rauert, C.; Pan, Y.; Okoffo, E. D.; O'Brien, J. W.; Thomas, K. V. Extraction and Pyrolysis-GC-MS Analysis of Polyethylene in Samples with Medium to High Lipid Content. *J Environ Expo Assess* **2022**, *1* (2), 13. https://doi.org/10.20517/jeea.2022.04.

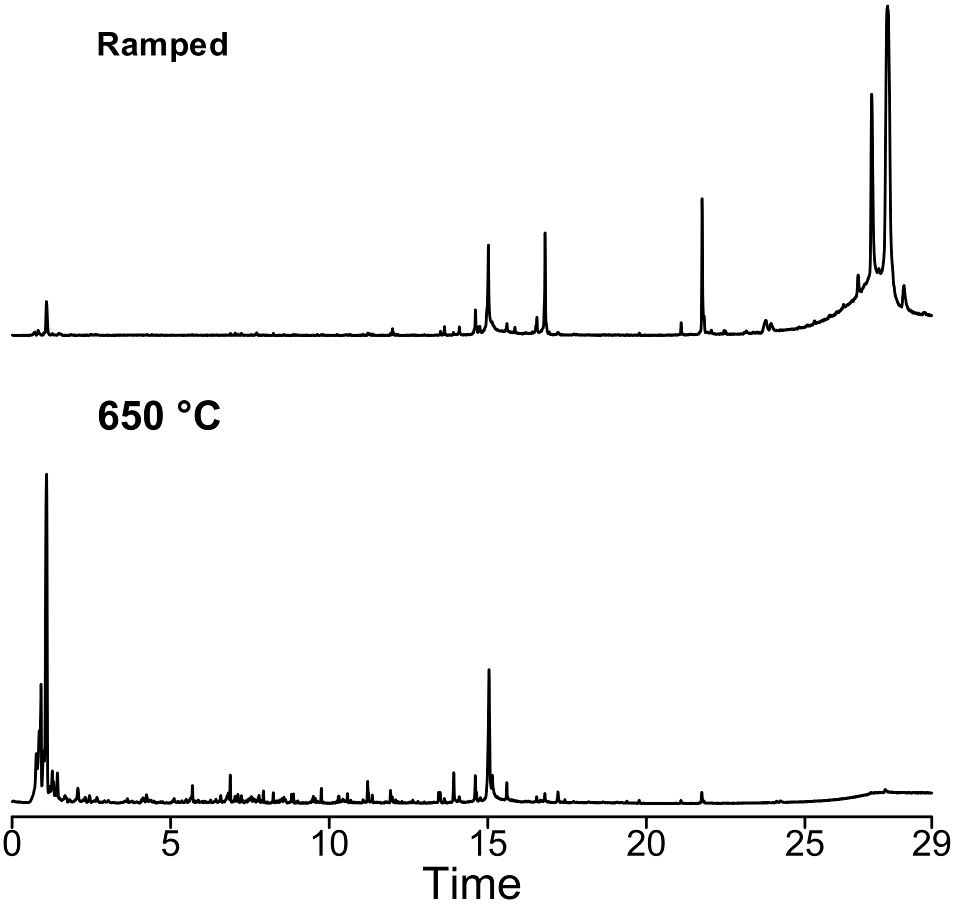

NIST-PPS analysis of the flash pyrolysis data gives a score for PE of 408 (23/48) as well as low scores for other polymers. A score of 0 (0/0) was returned for the ramped method. This is presumed to be due to some combination of time at temperature. With the ramped pyrolysis method, the GTO is mostly thermally desorbed before most degradation occurs (the purity of the GTO is not known, and thus, to what extent it is breaking down, even with the ramped pyrolysis method, is unknown).

Similarly, a sample of C94 was analyzed. The flash pyrolysis (650 °C) was run with a vent flow of 11mL/min during pyrolysis. This means the total flow through the pyrolyzer and inlet was significantly less than the ramped method, and the speed of the gas flow through the pyrolyzer was much slower. This is reflected in a significant increase in the alkadienes and a decrease in the high molecular weight pyrolyzates.

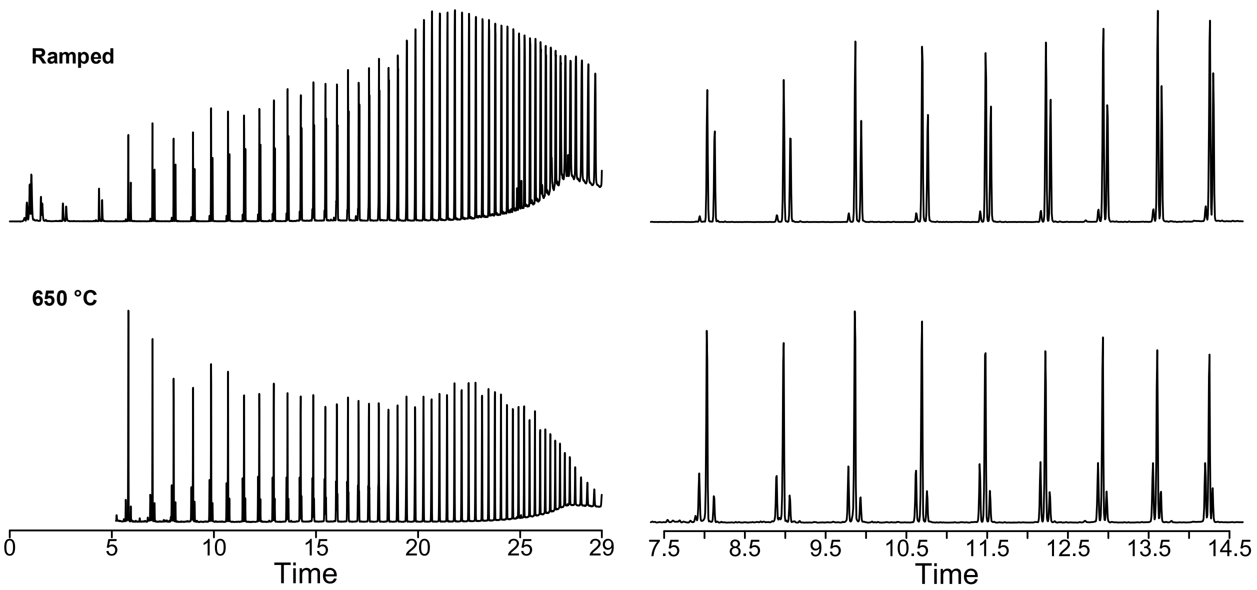

Note flash pyrolysis data employed a “solvent delay” of 5 minutes on the MS so no data was acquired before that time.

S6: Known issues running NIST-PPS (as of 9/2025):

1. Data file name: Due to the way AMDIS is called, if the data file has a space in it, AMDIS will not be called correctly, and the search app will hang.
2. If the results of the search end up with an empty results file, the search app will hang.

S7: Cosine similarity of combined mass spectra:

The combined mass spectrum of a pyrogram was made by using msAxel software to average over the whole pyrogram and background subtract in an area with no pyrolyzates generating a csv with “m/z” data in a column and “abundance” data in a column. In R the csv were read and the resulting spectra (a“m/z” vector and corresponding “abundance” vector as a list for both “query” and “library”) for the query and reference spectra were merged utilizing the following command:

merge(query, library, by = “m/z”, all = T)

and the cosine between the query and library abundance vectors was calculated utilizing the following command from the coop package:

cosine(queryabundancevector, libraryabundancevector, use = “complete.obs”)

This ignores the data if both spectra do not have a value at a particular m/z

Note this is different than the modified cosine metric of the match factor.

NIST Polymer Pyrolysis Search

*.d or netcdf file select

D:/pyrolysis/data/051425EI/PETPVCramp2-Centroid.cdf

Create RI calibration

Match Factor Cutoff

500 700 999

RI Penalty Rate

50 1,000

S/N Cutoff

3 50 1,000

RI cal file found

Polymer Search

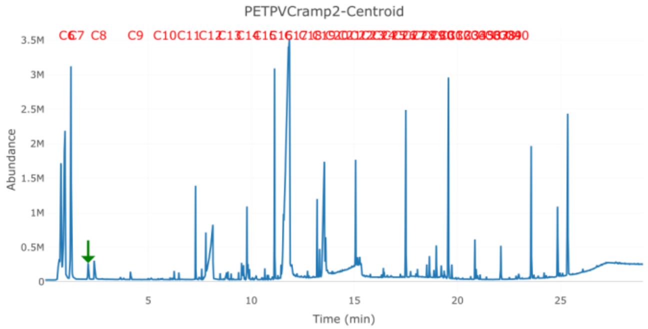

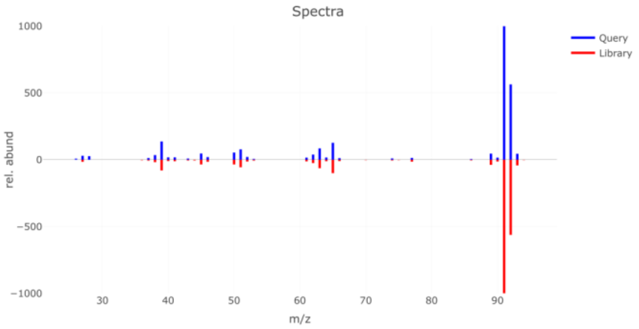

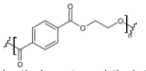

Polyethylene terephthalate

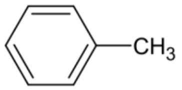

Show 10 entries Search:

polymer.list	count	libcount	meanmf	score
PET (8)	8	8	949.75	949.75
PVC (5)	5	5	936.33	936.33
PS (4)	2	4	955.87	477.94
PBT (9)	2	9	898.9	199.76

Showing 1 to 4 of 4 entries Previous 1 Next

Show 10 entries Search:

rt	ri	name	mf	formula	mw	dri	tol	mfp	modmf	polymer
1.2484	640.4	Benzene	956.7	C6H6	78	11.4	6.4	39.01	917.69	PVC (5); PBT (9); PET (8)
2.0855	752.9	Toluene	964.38	C7H8	92	6.5	7.53	0	964.38	PVC (5); PS (4)
4.139	886.1	Styrene	947.36	C8H8	104	6.6	8.86	0	947.36	PS (4)
6.2585	1037.9	Indene	926.72	C9H8	116	4.1	10.38	0	926.72	PVC (5)
7.2929	1129.3	Vinyl benzoate	957.14	C9H8O2	148	0.4	11.29	0	957.14	PET (8)
7.5962	1158.6	3-Methylindene	942.85	C10H10	130	4.7	11.59	0	942.85	PVC (5)
7.7928	1177.6	Naphthalene	930.03	C10H8	128	6.5	11.78	0	930.03	PVC (5)
8.1327	1211.6	Benzoic acid	942.37	C7H6O2	122	27.2	12.12	62.25	880.12	PBT (9); PET (8)
11.128	1561	Divinyl terephthalate	978.63	C12H10O4	218	0.4	15.61	0	978.63	PET (8)
11.8478	1656.7	4-((Vinyloxy)carbonyl)benzoic acid	964.84	C10H8O4	192	4.1	16.57	0	964.84	PET (8)

Showing 1 to 10 of 13 entries Previous 1 2 Next

S8: Screen shot of NIST PPS analysis of PET/PVC co-pyrolysis to show the different aspects of the app.
